# Genome-wide SNP analysis explains coral diversity and recovery in the Ryukyu Archipelago

**DOI:** 10.1038/srep18211

**Published:** 2015-12-10

**Authors:** Chuya Shinzato, Sutada Mungpakdee, Nana Arakaki, Noriyuki Satoh

**Affiliations:** 1 Marine Genomics Unit, Okinawa Institute of Science and Technology Graduate University, Onna, Okinawa 904-0495, Japan; 2 DNA Sequencing Section, Okinawa Institute of Science and Technology Graduate University, Onna, Okinawa 904-0495, Japan

## Abstract

Following a global coral bleaching event in 1998, *Acropora* corals surrounding most of Okinawa island (OI) were devastated, although they are now gradually recovering. In contrast, the Kerama Islands (KIs) only 30 km west of OI, have continuously hosted a great variety of healthy corals. Taking advantage of the decoded *Acropora digitifera* genome and using genome-wide SNP analyses, we clarified *Acropora* population structure in the southern Ryukyu Archipelago (sRA). Despite small genetic distances, we identified distinct clusters corresponding to specific island groups, suggesting infrequent long-distance dispersal within the sRA. Although the KIs were believed to supply coral larvae to OI, admixture analyses showed that such dispersal is much more limited than previously realized, indicating independent recovery of OI coral populations and the necessity of local conservation efforts for each region. We detected strong historical migration from the Yaeyama Islands (YIs) to OI, and suggest that the YIs are the original source of OI corals. In addition, migration edges to the KIs suggest that they are a historical sink population in the sRA, resulting in high diversity. This population genomics study provides the highest resolution data to date regarding coral population structure and history.

Coral reefs, which host one-third of all described marine species, are declining due to various anthropogenic insults, resulting in ocean acidification, seawater temperature increases, increased sediment, higher nutrient and pollutant loads, greater frequency and intensity of storms, etc^1^,^2^,^3^,^4^. Understanding coral genetic diversity, connectivity, and population structure is essential to increase the effectiveness of coral restoration efforts^5^,^6^,^7^. Corals exhibit a typical metapopulation structure with each population comprising many sub-populations connected by larval dispersal that occurs during the planktonic larval phase^8^. When a population is severely damaged, larvae supplied from undisturbed populations are essential for recovery^9^.

The southern Ryukyu Archipelago (sRA) has unique geographic, hydrodynamic, and historical features. It is located in southwestern Japan, spanning over 600 km (Fig. 1), and the strong Kuroshio ocean current runs from the eastern Philippines through the Archipelago (Fig. 1b). Coral reefs throughout the sRA are regarded as a marine biodiversity hotspot^10^. In 1998, a large percentage of *Acropora* corals at OI were lost as a result of a global coral bleaching event^11^,^12^, but they are now gradually recovering^13^,^14^. In contrast, the Kerama Islands (KIs), located approximately 30 km west of OI have retained healthy corals and high diversity, suffering only 6.7 to 23.4% mortality around Aka Island during the mass bleaching^15^, and maintaining high coral coverage (23–35%) from 2003 to 2007^14^. The Keramas became a Japanese National Park in 2014. Given the stark differences in the recent history of these geographically close populations, we set out to answer several questions. How have corals recovered at OI? How were the KIs able to retain such diverse, healthy coral populations? To what extent did KI corals contribute to the OI coral recovery? Next-generation sequencing (NGS) has allowed us to sample genomes much more densely and to observe the patterns of genetic variation that result from the full range of evolutionary processes acting across the genome^16^. Now it is possible to identify thousands of single nucleotide polymorphisms (SNPs) throughout a genome, and to acquire vast quantities of genomic information from a sample, whereas formerly it was only possible to examine small numbers of neutral molecular markers (e.g. allozymes, microsatellites) covering a very limited sampling of the genome. Today we can improve the precision of population genomic analyses by greatly increasing the number of markers examined. Recently, genome-wide SNP data have become widely used for studying population structure and historical migration patterns of various organisms^17^,^18^ including fishes^19^,^20^ and a seahorse^21^. Thus NGS technologies have become powerful tools in conservation ecology^16^.

The stony coral, *Acropora digitifera*, is widely distributed in the Indo-Pacific Ocean (Fig. 1a) and it is the only coral species with a decoded genome^22^. *A. digitifera* is a broadcast-spawning coral and typically inhabits the shallow, flat reef zone in Okinawa (Fig. 1a). Waves and ocean currents should significantly influence larval dispersal. Larval culture experiments with *A. digitifera* have shown that the maximum settlement competency period of larvae, the period within which larvae can settle on a suitable substrate, is 45 days^23^. An investigation of ocean current flow between the KIs and OI using high-frequency radar and GPS buoys showed that coral larval could drift from the KIs to OI in 3 to 4 days, implying that the KIs are an important larval source for the west-coast of OI^24^. Moreover, a study of *A. digitifera* population genetics using microsatellite markers suggested high genetic connectivity across the entire RA^25^. However a detailed understanding of population structure was nonexistent. Using the decoded genome of *A. digitifera*, we performed the first genome-wide SNP analysis of corals and succeeded in mapping detailed population structure and migration patterns of *A. digitifera* in the sRA.

## Results and Discussion

We first improved the original *A. digitifera* genome assembly^22^. The new version (1.1) is approximately 447 Mbp and comprises 2,420 scaffolds with an N50 of 484 Kbp (Supplementary Table S1). Longer-range continuity of the genome assembly and an increase in the N50 scaffold size from 191 kbp to 484 kbp (Supplementary Table S1), allowed us to assess long-range linkage disequilibrium. In addition, the total nucleotide count without ambiguous bases (gaps) increased from 365 Mbp to 378 Mbp, indicating that the new reference genome could produce more SNP data than the previous version. Thus we concluded that the quality is significantly improved compared with the original release^22^.

**Figure 1:**
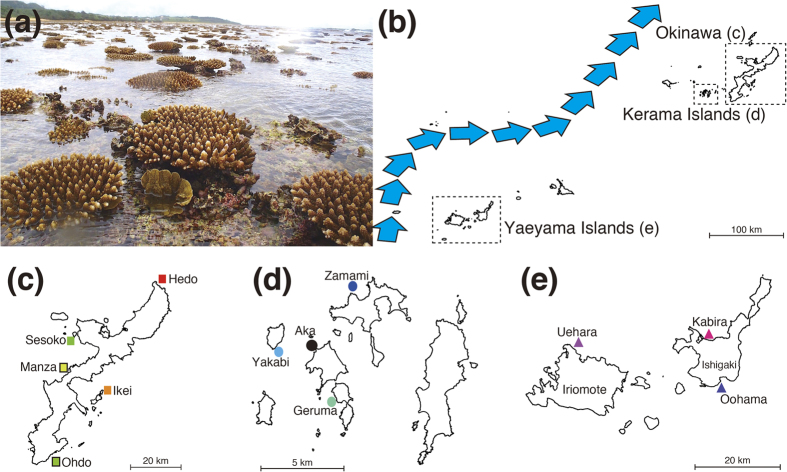
A scleractinian coral, *Acropora digitifera,* and sampling locations in the southern Ryukyu Archipelago. (**a**) Typical habitat of *A. digitifera* colonies used in this study. Some colonies are exposed to air at low tide. Photo taken at Uehara, Iriomote Island. (**b**) Overview of Okinawa prefecture. Blue arrows indicate the Kuroshio Current in May 2014, based on the Japan Meteorological Agency website (http://www.jma.go.jp/jma/indexe.html). (**c**) Sampling sites at Okinawa Island, (**d**) the Kerama islands, (**e**) the Yaeyama islands (Ishigaki and Iriomote islands are identified). A shape file for Okinawa prefecture was downloaded from National Land Numerical Information, Japan (http://nlftp.mlit.go.jp/ksj/gmlold/index.html) and was visualized as maps using maptools in R (version 3.1.1)^31^. All maps are oriented with North at the top.

We collected a total of 155 individual colonies from OI, the KIs and the Yaeyama Islands (YIs) (Fig. 1b–e). Genomes of all samples were re-sequenced and SNPs of each sample were identified. We excluded individuals with heterozygosity rates >0.12 and/or missing genotype rates >0.35 (Supplementary Fig. 1). Subsequent removal of SNPs that showed extensive deviation (p < 0.001) from Hardy-Weinberg equilibrium (HWE), with a call rate less than 99% and with a very low minor allele frequency (threshold of 2%), yielded 905,561 SNPs. After removal of related sample outliers, 122 individuals from 11 locations remained (all individuals from the Aka site were removed), and these were used for subsequent analyses.

Linkage disequilibrium decayed within <10 kbp at all sampling locations and we could not observe different decay patterns between sampling locations (Supplementary Fig. 2), indicating no population-specific bottlenecks in these areas. We tried to estimate individual ancestries of *A. digitifera* populations in the sRA using model-based clustering methods, ADMIXTURE^26^ and fastSTRUCTURE^27^ (for an example, see Supplementary Fig. 3), but we failed to detect clear population structure. In both software packages, the appropriate number of populations (K) to best explain acroporid genetic differentiation was K = 1 (K can range from 1 to 10), suggesting that there is a single population of *A. digitifera* in the sRA.

**Figure 2:**
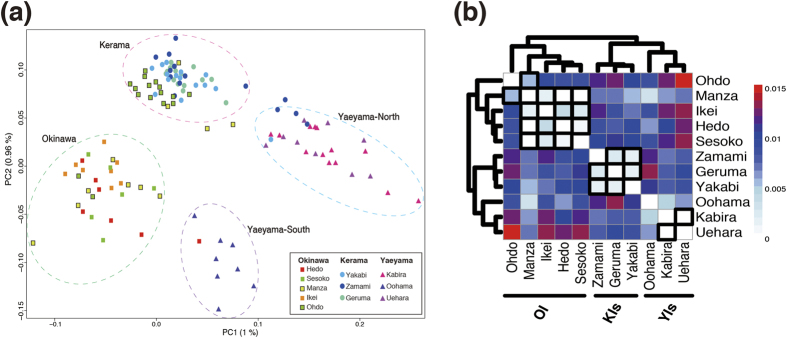
Principal components analysis and genetic distances and suggest the existence of four clusters in the sRA, located in Okinawa, the KIs, Yaeyama North, and Yaeyama South. (**a**) Principal components analysis (PCA) of the filtered dataset of 122 individuals and 905,561 SNPs after trimming of related samples with smartrel and outliers with smartpca. Tracy-Widom statistical significance of PC1 and PC2 was <1e^−35^. Individuals from Okinawa Island, the KIs, and the YIs are shown with squares, circles, and triangles, respectively. Four subpopulations, Okinawa, Kerama, Yaeyama-North, and Yaeyama-South are circled in green, pink, blue, and purple dotted lines, respectively. (**b**) Heatmap showing pairwise *Fst* values based on Weir and Cockerham weighted estimates between sampling sites. All points were clustered by pairwise *Fst* values, based on the Complete Linkage Clustering method. Site combinations without boxed bold lines indicate significant differences (p-value < 0.001, ANOVA statistics across 10 eigenvectors in PCA).

Although model-based clustering methods for estimating ancestry could not detect clear population structure, principle component analysis (PCA) resolved the 122 individuals into four clusters, such that individuals from the same island or close locations tended to fall into the same cluster. Thus we named these Okinawa, Kerama, Yaeyama-North, and Yaeyama-South, respectively (Fig. 2a). The first two principal components explained 1% and 0.96%, respectively, of the total variance among SNP data of 122 individuals. Two distinctive clusters in YI possibly originated from larval dispersal interference caused by Sekisei lagoon, the largest coral reefs in Japan, located between Ishigaki and Iriomote islands. Most individuals from the KIs (Yakabi, Geruma and Zamami) were clustered together. Individuals from three points in Okinawa (Oku, Ikei, and Sesoko) were observed exclusively in the Okinawa cluster (Fig. 2a). On the other hand, a large number of individuals from Ohdo and some individuals from Manza were observed in both the Kerama and Okinawa clusters (Fig. 2a). This suggests that recent migration from the KIs to OI (mainly south coast, some west coast) has occurred and that these might be first-generation migrants. In addition, some individuals in the KIs (mainly Zamami) had SNP profiles similar to those of the Yaeyama-North cluster, and an individual from Hedo (the northern tip of Okinawa) was grouped with the Yaeyama-South cluster (Fig. 2a), suggesting recent migration events between the KIs and Yaeyama-North and Okinawa and Yaeyama-South, respectively.

Slight genetic differentiation estimated by pairwise *Fst* values for all site combinations (–0.0007 to 0.0138) also suggested that there is a single population in the region. Although low *Fst* values were estimated for all combinations, sampling locations on the same island or close locations were clustered together (Fig. 2b). Previous coral population genetics studies using allozymes and microsatellites reported no correlation between genetic connectivity and geographic distance in *A. digitifera* in the sRA^23^,^25^. However we detected a significant isolation-by-distance correlation at the geographic scale of the study (P < 0.001 and R^2^ = 0.3715; Supplementary Fig. 4). ANOVA statistics across 10 eigenvectors in PCA (Fig. 2a) also revealed that sampling locations that were not significantly different (P > 0.001) are clustered on the same island or in proximate locations (Fig. 2b), as in the case of *Fst* values. Previous studies suggested that high genetic connectivity exists across the 1,000 km length of the RA^25^ and across the 1,500 km distance between the RA and the Ogasawara Islands. Those results implied that long-distance dispersal of *Acropora* larvae between high-latitude islands in Japan^28^ might be frequent; however, the larger dataset in this study makes it clear that this is not the case.

This is neither surprising, nor is it an isolated example. In *Seriatopora hystrix,* as in other brooding corals, the majority of larval recruitment in northern Western Australia occurred within 100 m of the natal colony^29^ and long-distance dispersal among high-latitude reefs in eastern Australia rarely occurred^30^. Even in cases of broadcast spawners, it is reported that two coral species possessing contrasting reproductive modes (*Goniastrea favulus*: sticky, negatively buoyant eggs and larvae, *Platygyra daedalea*: positively buoyant egg-sperm bundles) are genetically subdivided by distances as small as 50–100 m in the southern Great Barrier Reef^31^. A majority of *Montipora capitata* colonies are derived from self-recruitment across the Hawaiian Archipelago^32^. Although local biological, ecological, geographic factors, and ocean currents could have great influence on larval dispersal and coral recruitment, long-distance migration may be less frequent than suggested and may not occur in every generation. Instead, self-recruitment may be the major source of larval recruitment of *Acropora* corals in the sRA and may play an important role in coral recovery recently observed in OI^14^.

**Figure 3:**
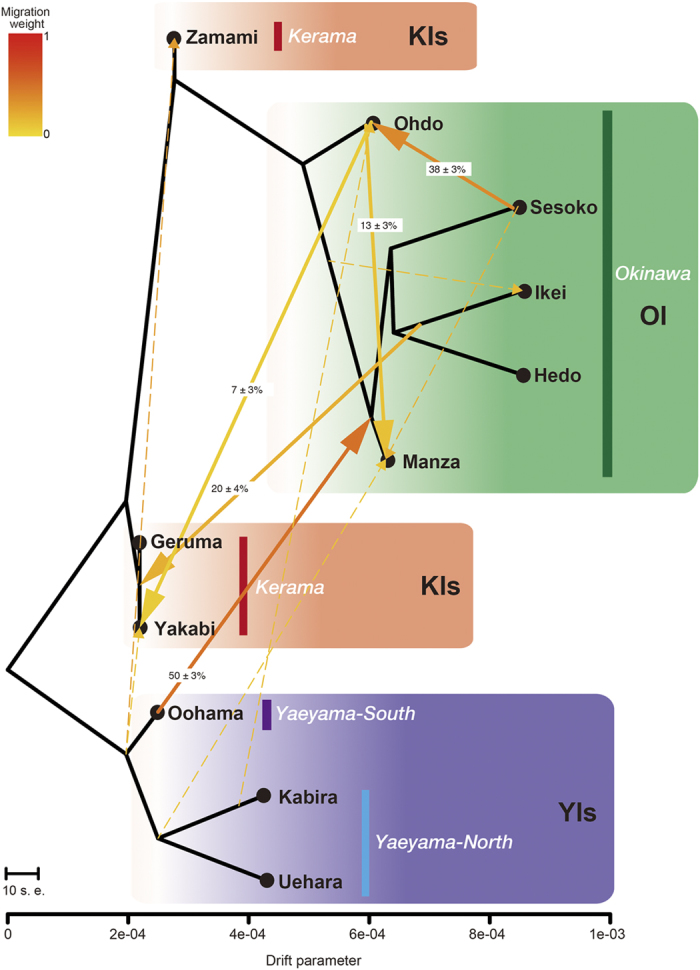
Migration patterns of *A. digitifera* in the sRA, showing that the YI populations function as source populations for OI, while the KIs constitute a historical sink. Inferred tree of *A. digitifera* populations in the sRA with thirteen migration events. Migration arrows are colored according to their weight. The migration weight represents the fraction of ancestry derived from the migration edge. Migration edges with p-value estimated by jackknife to be below 0.01 are shown. Migration edges with bootstrap support less than 50% are shown with dotted lines. Horizontal branch lengths are proportional to the amount of genetic drift that has occurred in each branch.

How were the coral reefs established, and how have population structures been maintained? We constructed admixture graphs using *TreeMix* to examine the population history of *A. digitifera* in the sRA. The maximum-likelihood population tree inferred without admixture events was similar to the clustering result obtained with *Fst* values (Fig. 2b), clearly correlating genetics with island locations (Supplementary Fig. 5a). Next we sequentially allowed for 0 to 15 migration events in the *TreeMix* analysis (Fig. 3). The increase in likelihood beyond thirteen such events was marginal, and stepwise comparisons of log likelihood between migration events became insignificant between 13 and 14 events (likelihood-ratio test, p > 0.05) (Supplementary Fig. 6). Thus we chose thirteen migration events for the *TreeMix* analysis. Five migration edges had more than 50% bootstrap support (Fig. 3, Supplementary Table 4). We detected strong long-distance migration spanning 400 km, from the YIs to OI. Individuals from Oohama, Ishigaki Island, and Hedo, Sesoko, Ikei, and Manza in Okinawa share 50 ± 3% ancestry (Fig. 3). Three-population tests also supported such migration (Supplementary Table 3). These suggest that the *A. digitifera* population in the sRA is partially maintained by long-distance migrations across the sRA; thus low genetic distance (*Fst* values, Fig. 2b) and common ancestry estimated by model-based clustering methods are observed in this study (Supplementary Fig. 3).

Complex migration patterns within OI were further inferred: migrations from Sesoko to Ohdo, and Ohdo to Manza (Fig. 3), and migrations from OI to the KIs were also detected (Ikei to Geruma and Yakabi, and Ohdo to Yakabi, Fig. 3). Three-population tests indicated active migrations from a variety of locations to Yakabi Island (KIs) (Supplementary Table 3). Although no individuals from Ikei were detected in the Kerama cluster using PCA (Fig. 2a), a migration from Ikei to the KIs was detected (Fig. 3, Supplementary Table 3). This suggests that coral connectivity from the east coast of OI to the KIs is possible. Although bootstrap support is poor (<50%, Supplementary Table 4), we detected migrations from the YIs to the KIs (Fig. 3). Heterozygosity of the KIs, especially Geruma and Yakabi, are significantly higher than those of OI and the YIs (Supplementary Fig. 7). Although maintenance of high coral coverage in the KIs^14^ would explain the high heterozygosity in that area, the heterozygosity might also reflect past migrations from both OI and the YIs to the KIs. Low heterozygosity of OI and the YIs might reflect a recent population bottleneck caused by a significant decrease of *Acropora* coral coverage after the 1998 bleaching event^11^,^12^,^33^.

Previous research using high-frequency radar and global positioning system buoys to study the length of the *Acropora* larval settlement competency period, suggested that coral larval transport had occurred from the KIs to OI^24^. In 1998, a massive bleaching event occurred at OI^11^ and after a decade, *Acropora* colonies slowly recovered along the west coast of Okinawa^14^, including Sesoko^13^. Van Woesik *et al.* (2011) proposed that the KIs facilitated *Acropora* recovery on Sesoko by supplying recruits. However the present PCA analysis did not suggest that any Sesoko individuals came from the Kerama cluster (Fig. 2a) and no migration from Kerama to Sesoko was supported by *TreeMix* or the three-population analysis (Supplementary Table 3). Thus it is improbable that the KIs were a major source for *Acropora* recovery in OI. A migration to the south coast of OI (Ohdo) was suggested by PCA (Fig. 2a); however, it is apparent that the KIs are a far more limited larval source than previously thought. Instead it seems that coral recovery in Sesoko occurred via recruitment from local sources that survived the bleaching event, or that it regrew from cryptic remnant tissues^34^,^35^. Conversely our results of *TreeMix* and three-population test showed that the YI populations have a strong influence on OI populations (Fig. 3). Recent long-distance migration, especially from Ishigaki to OI, might explain the low genetic distances (*Fst*) (Fig. 2b) detected in this study.

In conclusion, this first coral population genomics study, analyzing genome-wide SNP data, has considerably illuminated the recent history of acroporid coral population structure in the sRA, even though genetic distances are quite low in the area. (1) Coral recovery observed in the last decade in OI has been accomplished mainly by self-recruitment. Because of low gene flow between locations, local conservation and restoration efforts will be required for each coral reef in the sRA. (2) Migrations from both OI and the YIs to the KIs were documented and suggest that the KIs are a “historical sink” of coral connectivity in this region, maintaining high coral diversity. (3) The YI populations have historically influenced the establishment and maintenance of OI populations. These findings, resulting from the high resolution of next-generation sequencing technology, provide data that will be essential for future coral reef conservation in the region.

Although re-sequencing-based genome-wide SNP analyses require reference genomes and are restricted to species with decoded genomes, now reference-free methods for identifying genome-wide SNPs are available (e. g. RAD-seq^36^). Application of RAD-seq to coral population genetics^37^ and linkage mapping^38^ have been reported; however, the number of SNPs identified was much smaller than by the whole genome re-sequencing method used in this study. Re-sequencing-based, genome-wide SNP data enable us to obtain large amounts of biological information, and will become an important and powerful tool for coral biology.

## Methods

### Sample collection

Tissue samples were collected from 155 individual *Acropora digitifera* colonies in the southern RA from 2011 to 2014 under Okinawa Prefectural Permits (Numbers: 22–29, 23–34, 24–48, 25–67). Collection sites include five locations (Hedo, Sesoko, Manza, Ikei, and Ohdo) on Okinawa Island, four locations (Geruma, Yakabi, Zamami, and Aka) in the Kerama Islands, two locations (Oohama and Kabira) near Ishigaki Island, and one (Uehara) at Iriomote Island in the Yaeyama Islands (Fig. 1). We identified *A. digitifera* in the reefs and used colonies showing typical corymbose shape (Fig. 1a). As *A. digitifera* corals in the RA are often found around reef-edges, most samples were collected at depths of 1–2 m, where some corals are exposed to the air during low tide (Fig. 1a). All Kerama samples came from slightly deeper reefs (1–4 m). To avoid duplicate collections of colonies that could have been produced through asexual fragmentation or propagation, only colonies that were physically distinct and at least 5 m from other colonies were sampled. A single branch about 1–1.5 cm long was taken from each colony using a nipper. Samples were preserved in the guanidinium reagent, CHAOS^39^, and stored at room temperature.

### DNA isolation and genome re-sequencing

Coral branches in CHAOS buffer were incubated at 56 °C for 48 hours. After that, coral skeletons were removed and preserved in the event that future species confirmation using skeletal characters might be needed. After ethanol precipitation, pellets were dissolved in ALT buffer from a QIAGEN DNeasy blood and tissue kit (cat. 69504) and then they were digested with proteinase K for 48 hours at 56 °C. Samples were further purified according to QIAGEN kit instructions, including RNaseA treatment. At the end, DNAs were eluted from spin columns using AE buffer and kept at 4 °C. Library preparation for re-sequencing of DNA from each individual genome using Illumina platforms (500 bp insert size) was done according to the manufacturer’s instructions. Libraries were sequenced using an Illumina Genome Analyzer IIx (GAIIx) (75 bp paired-end reads) and Hiseq 2000 (100 bp paired-end reads). Raw sequence data were submitted to the DNA Databank of Japan (DDBJ) Sequence Read Archive (DRA) under accession number DRA003938 (BioProject PRJDB4188).

### SNP identification and detection

To improve assembly of the reference genome of *A. digitifera*, we used sequence data from an individual from Hedo, Okinawa, reported in Shinzato *et al.* (2011)^22^, and further scaffolding was performed with SSPACE (ver. 1.1)^40^. Gaps inside the scaffolds were closed using GapCloser (ver. 1.10)^41^. To overcome potential assembly errors arising from tandem repeats, sequences that were aligned to another sequence over 50% of their lengths with BLASTN (e-value: 1e^−50^) were removed from the assembly. The genome assembly has been deposited with the DDBJ under project accession BACK02000001–BACK02054400 (contigs) and DF970692–DF973111 (scaffolds).

All Illumina reads from each individual were mapped to the reference sequence using BWA version 0.5.9^42^. We applied a soft-clipping base quality threshold of 10 (phred scaled) to avoid low quality bases in alignments, but otherwise used default settings. Unmapped reads and duplicated reads were removed using SAMtools version 0.1.18^43^. When reads map to multiple locations in the genome, BWA randomly assigns the hit to one location, with a mapping score of zero. Therefore, such reads are taken into account in coverage estimates, but the low mapping score downplays their effect on downstream variant calling. Results for each individual were enhanced by local realignment using the GATK toolkits version 2.7^44^ and duplicate-marked with Picard version 1.6 (http://picard.sourceforge.net).

Initial variant site identification was performed using low-coverage, multi-sample SNP calling with SAMtools mpileup and GATK with default settings from the realigned bam file. Consensus calls (~17.8 million SNPs) from both software packages were further filtered by including only no-missing-call SNPs and used as a known site for base quality recalibration using GATK with default settings. The resulting recalibrated bam files were then used as new input for variant calling with GATK. Then the highest scoring 10% of consensus calls (~75,000 SNPS) were used as a training set for variant quality recalibration and filtering with GATK.

### Data quality control and markers

Generally we followed the protocol for data quality assessment and control that is typically employed during genome-wide case-control association studies^45^. Samples with low DNA quality or concentration often have below average call rates and genotype accuracy. Therefore, the genotype failure rate and heterozygosity rate per individual are both measures of DNA sample quality. We inspected the distribution of mean heterozygosity across all individuals to identify those with an excessive or reduced proportion of heterozygous genotypes, which could indicate DNA sample contamination, fragmentation, or DNA from a different *Acropora* species. First, missing genotype and heterozygosity rates of each individual were calculated using Plink, version 1.9^46^. Observed heterozygosity rates per individual were calculated using the formula (N − O)/N, where N is the number of non-missing genotypes and O the observed number of homozygous genotypes for a given individual^45^. We used the Tukey-Kramer test to check differences in heterozygosity rates among all site combinations. Since the smallest number of samples from any sampling site was six (Sesoko), we used six individuals from each point to calculate pairwise linkage disequilibrium, using no missing SNP data with Plink. To avoid duplicated or related individuals, only independent SNPs were included in the analysis. SNPs exhibiting extended linkage disequilibrium (LD) in which a correlated (r^2^ = 0.2) pair of SNPs within a window of 50 kb, were removed from the data set using Plink. We excluded markers that showed extensive deviation (p < 0.001) from HWE, with a call rate ≤99%, a missing rate of 1%, and with a very low minor allele frequency (MAF) (threshold of 2%) using Plink. We used this SNP data set for all analyses in this study. Pruned data were subjected to smartrel from EIGENSOFT (version 5.0.2)^47^,^48^ to identify related samples and smartpca was used to identify outliers, to perform Principal Component Analysis (PCA), and to assess significant differences between sampling points by ANOVA across eigenvectors.

### *Fst* analysis

*Fst* values were calculated for bi-allelic sites using the method of Weir and Cockerham with vcftools version 0.1.12b^49^. *Fst* values were visualized with a heatmap and clustered using the complete linkage method for hierarchical clustering using the R package “pheatmap” (http://cran.r-project.org/web/packages/pheatmap/index.html). A plot of isolation-by-distance was used to explore the relationship between the calculated geographic distance and (1-*Fst*)/*Fst*, as a genetic differentiation index. The log of coastal distances between sampling locations (km) was determined using Google Earth. IBD significance was tested using Pearson’s correlation (R,version 3.1.1)^50^.

### *TreeMix* and admixture analyses

In order to estimate historical relationships among populations, we used *TreeMix* version 1.12^51^. For all analyses we used an SNP window size (–K) of 1,000. This corresponds to a window size of approximately 0.46 Mbp (the expected genome size of *A. digitifera* is 420 Mbp and a window size of 1,000 divided 905,561 SNPs into 181 blocks), which far exceeds the known extent of LD (see Supplementary Fig. 2) in *A. digitifera*. *TreeMix* was run using the –global option which corresponds to performing a round of global rearrangements of the graph after initial fitting. In addition, 100 bootstrap replicates were performed using the –bootstrap option for numbers of migration events ranging from 3–14 (–m option) to assess statistical support for certain migration events. We performed a likelihood-ratio test for stepwise comparison of log likelihood between migration events. The three-population test is a formal test of admixture that provides clear evidence of admixture, even if the gene flow events occurred hundreds of generations ago^52^; when f3(X;A,B) is negative, deviation from “treeness” is detected and X appears to be a mixture of A and B. For the three-population test, we used the *TreeMix*-implemented three-population test^53^. We used all combinations of three points out of the 11 sampling sites.

## Additional Information

**How to cite this article**: Shinzato, C. *et al.* Genome-wide SNP analysis explains coral diversity and recovery in the Ryukyu Archipelago. *Sci. Rep.* **5**, 18211; doi: 10.1038/srep18211 (2015).

## Supplementary Material

Supplementary Information
