# Supplementary material for: Genome-wide SNP analysis explains coral diversity and recovery in the Ryukyu Archipelago
Source: Sci Rep. 2015 Dec 10;5:18211. doi: 10.1038/srep18211 (PMC4674749; doi:10.1038/srep18211)
Supplement: Supplementary Information [file srep18211-s1.pdf]

Genome-wide SNP analysis explains coral diversity and recovery in the Ryukyu Archipelago

Chuya Shinzato[1*,#], Sutada Mungpakdee[1*], Nana Arakaki[2], Noriyuki Satoh[1]

[1]Marine Genomics Unit, Okinawa Institute of Science and Technology Graduate University, Onna, Okinawa 904-0495, Japan

[2]DNA Sequence Section, Okinawa Institute of Science and Technology Graduate University, Onna, Okinawa 904-0495, Japan

----

*These authors contributed equally to this work.

[#]Corresponding author. Phone: +81-98-966-8653; Fax: +81-98-966-8653

E-mail: c.shinzato@oist.jp

Supplementary Figure Legends

Supplementary Fig. 1. Heterozygosity rate and missing rate for the 155 individuals. The heterozygosity rate cutoff of 0.12 and the missing genotype rate of 0.35 are shown with dashed lines. Colors represent sampling sites as follows; blue: Hedo, green: Oodo, red: Ikei, blueviolet: Uehara, dark olive green: Ishigaki island (Kabira, Oohama), magenta: Kerama, pink: Manza, brown: Ohdo, gray: Sesoko.

Supplementary Fig. 2. Linkage disequilibrium patterns of 11 sampling sites showing that there are no differences in decay patterns. The X axis shows pairwise distances of SNPs and the y axis shows average r^2 in non-overlapping 100 bp bins.

Supplementary Fig. 3. An example of ancestry estimation using model-based clustering ADMIXTURE (K=4). Cluster names identified by PCA (Fig. 2a) are shown. Pie charts show average proportions of ancestral populations in each cluster.

Supplementary Fig. 4. Isolation-by-distance (IBD) estimated from pairwise *Fst* / (1-*Fst*) values for combinations of 11 sampling sites, showing that the log of genetic distance is positively correlated with geographic distance.

Supplementary Fig. 5. Inferred tree of *A. digitifera* populations in the sRA without admixture events, showing that the tree topology is clearly related to island biogeography. (A) Maximum likelihood tree generated by *TreeMix*. (B) Pairwise residual fit generated by *TreeMix*. Residuals above zero represent populations that are more closely related to each other in the data than in the best-fit tree, and thus are candidates for admixture events. Colors are described in the palette on the right.

Supplementary Fig. 6. Increase in log-likelihood as a function of the number of migration events (0 to 15) in *TreeMix*, showing that a value of 13 migration events (used in Figure 3) is reasonable. Asterisks indicate the migrations that stepwise comparisons of log likelihood between migration events (13 and 14, and 14 and 15) became insignificant (likelihood-ratio test, $p > 0.05$).

Supplementary Fig. 7. Heterozygosity rate at each sampling site, showing that the KIs, with high heterozygosity, constitute a sink population. The X-axis represents sampling sites and the Y-axis represents heterozygosity rate per individual. The median, the lower and upper quartiles, and outliers are shown. Colors are allocated to sub-populations identified in the PCA analysis (Okinawa: green, Kerama: pink, Yaeyama-North: blue and Yaeyama-South: purple). Tukey-Kramer test was performed for checking differences in heterozygosity rates among all site combinations. Asterisks show significant differences between sites (p-value below 0.01).

Supplementary Table legends

Supplementary Table 1. Summary of the *Acropora digitifera* genome assembly version 1.1.

Supplementary Table 2. Mapping coverage for each individual.

Supplementary Table 3. Results of three-population test. Significant migration (Z score below -1.96, $p < 0.05$) results are shown.

Supplementary Table 4. Migration weight and p-values inferred by *TreeMix* with 13 migration events allowed. Bootstrap support more than 50% (see Supplementary Table 5) are shown.

Supplementary Table 5. Bootstrap support percentages (100 replicates) for each migration edges (m= 3 to 14).

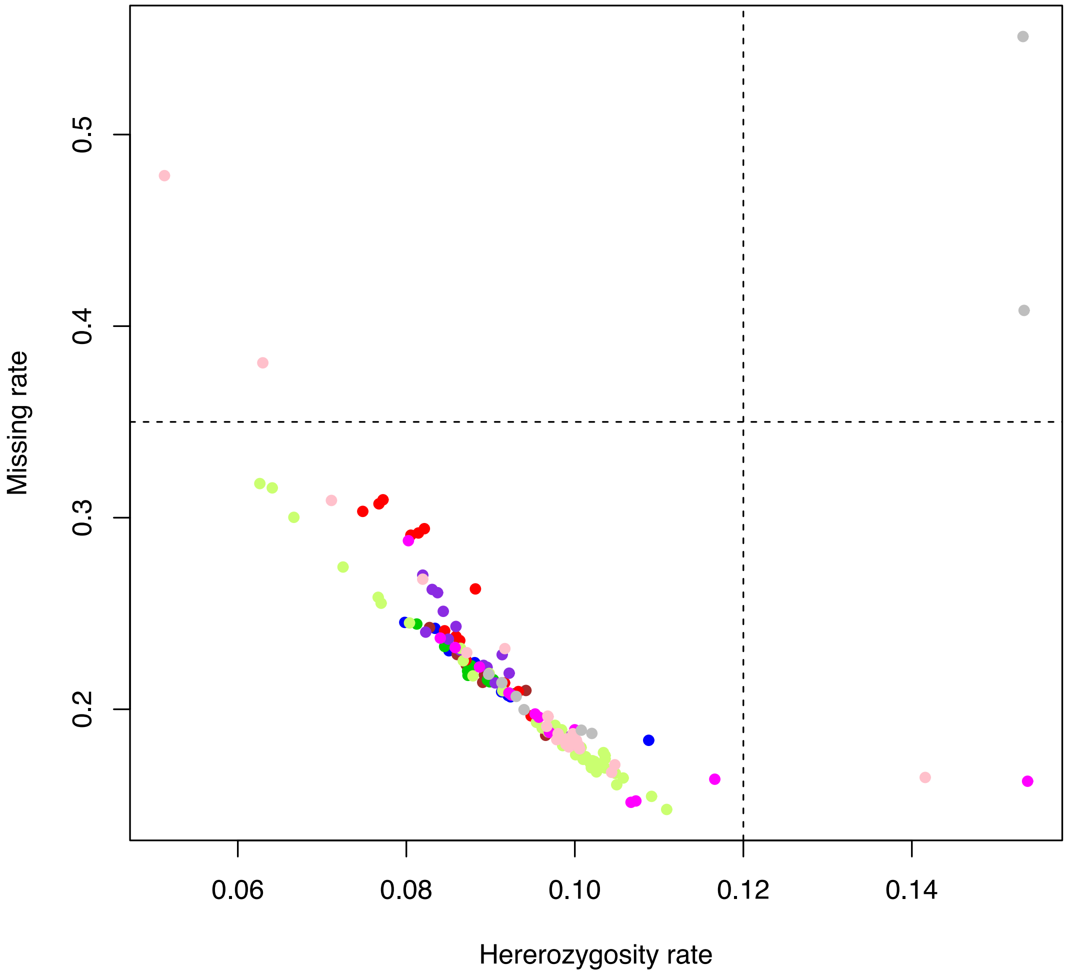

Supplementary Fig. 1

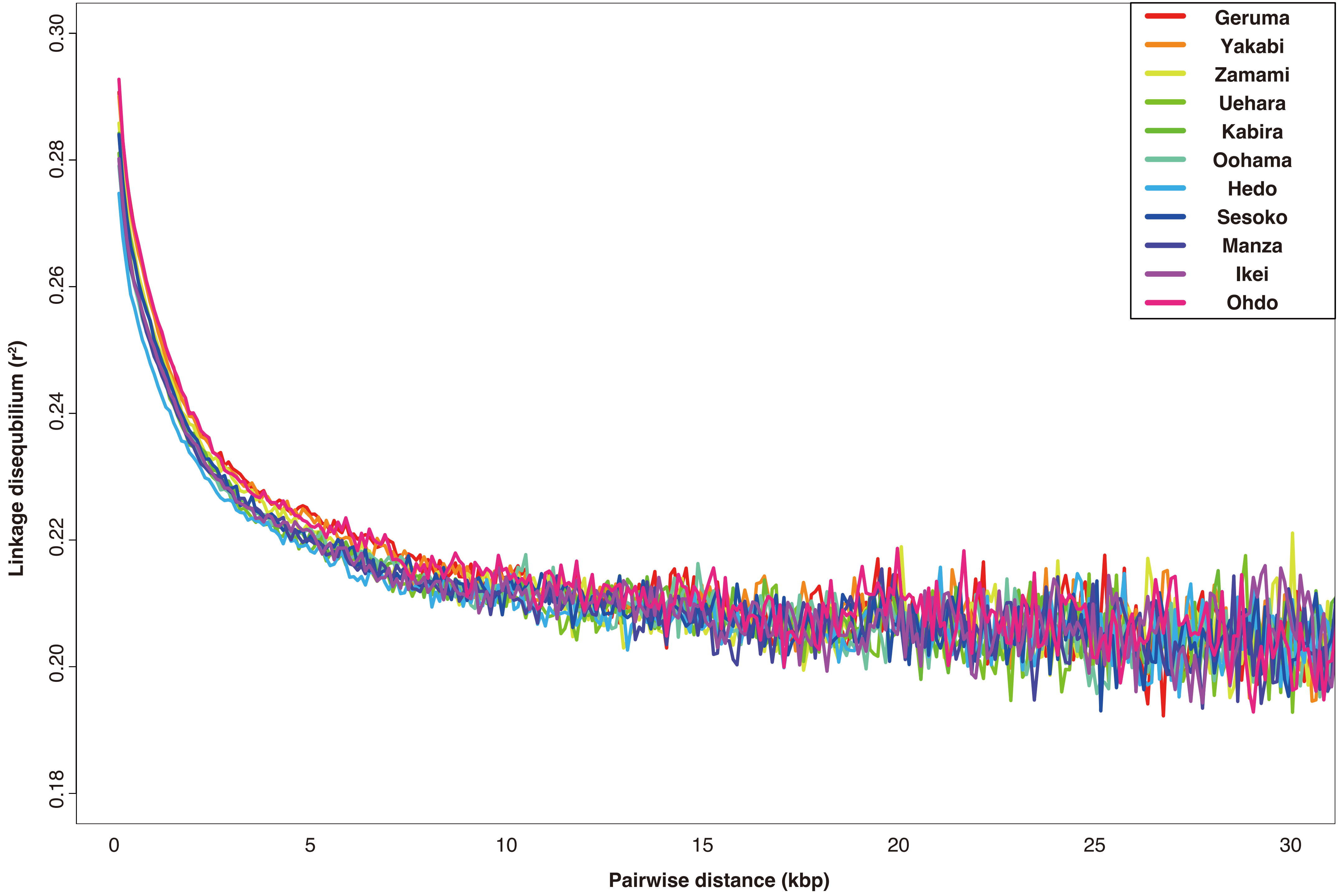

Supplementary Fig. 2

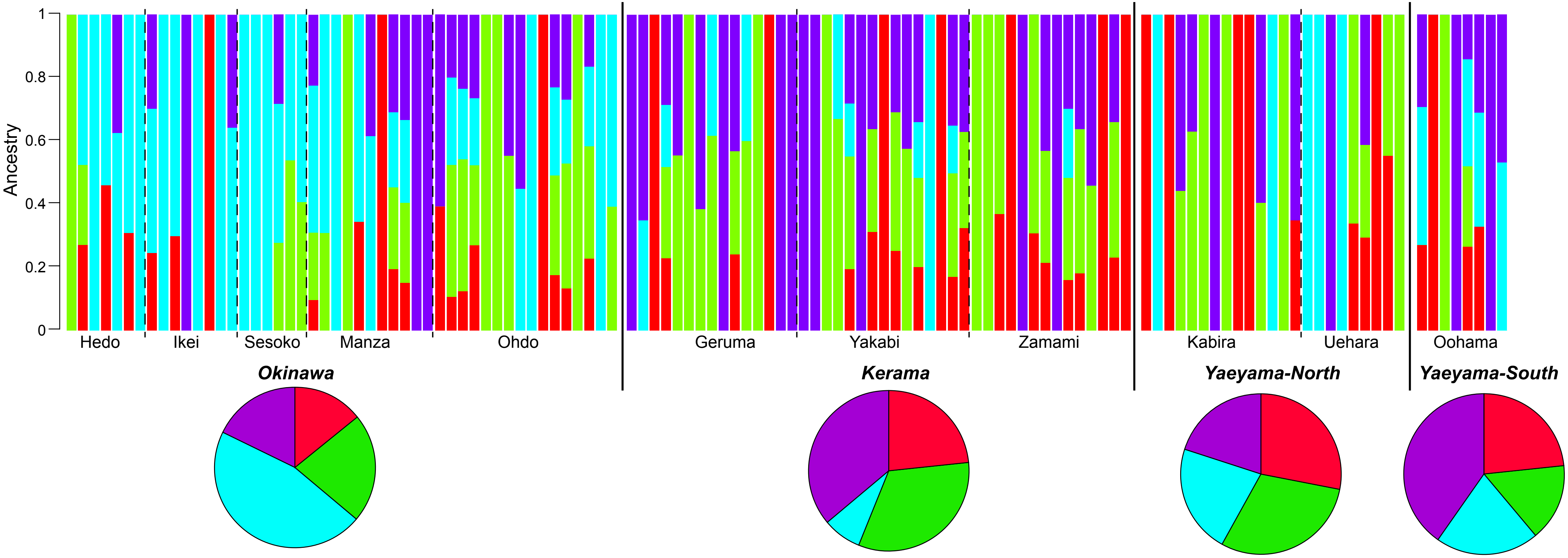

Supplementary Fig. 3

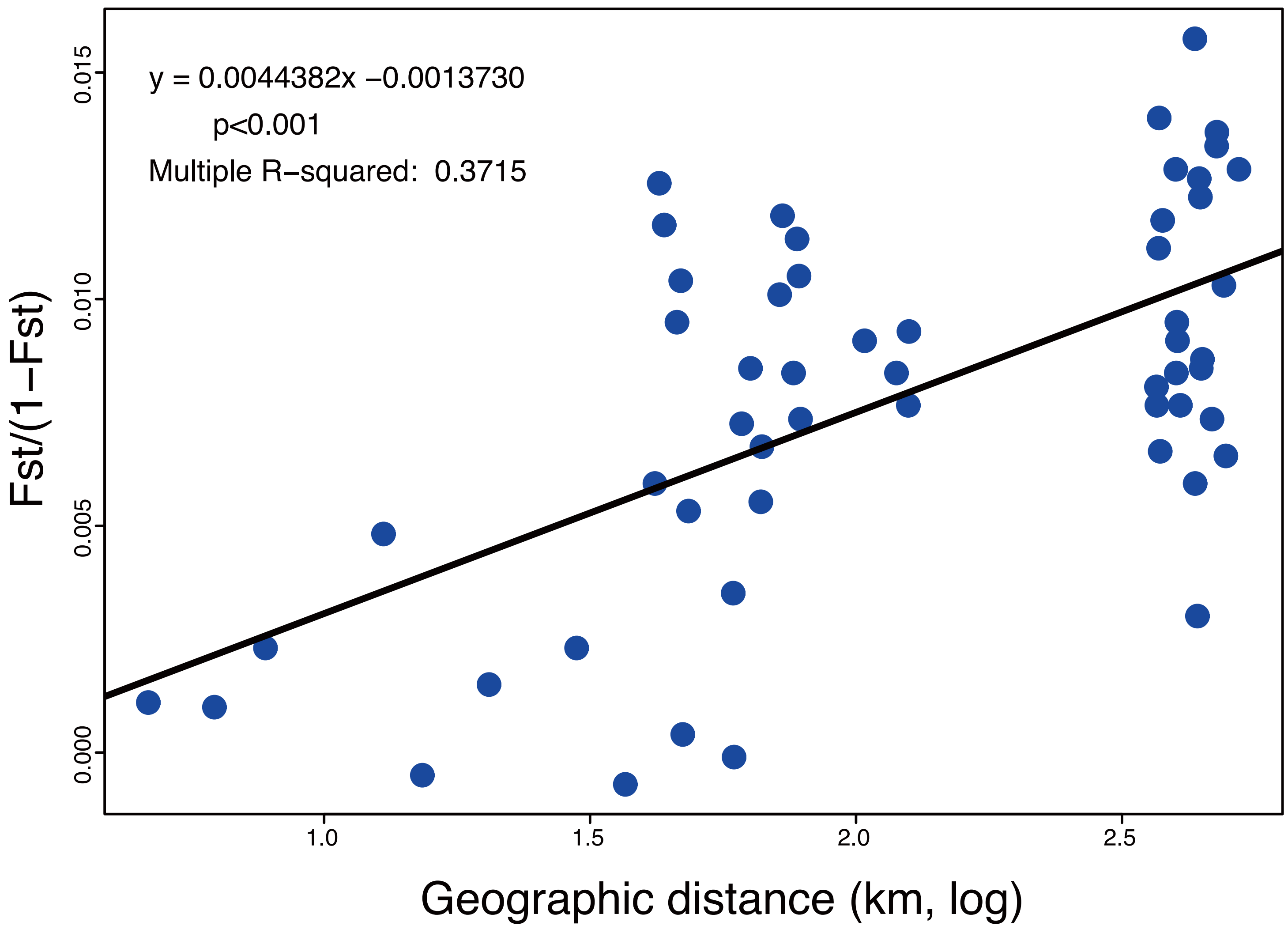

Supplementary Fig. 4

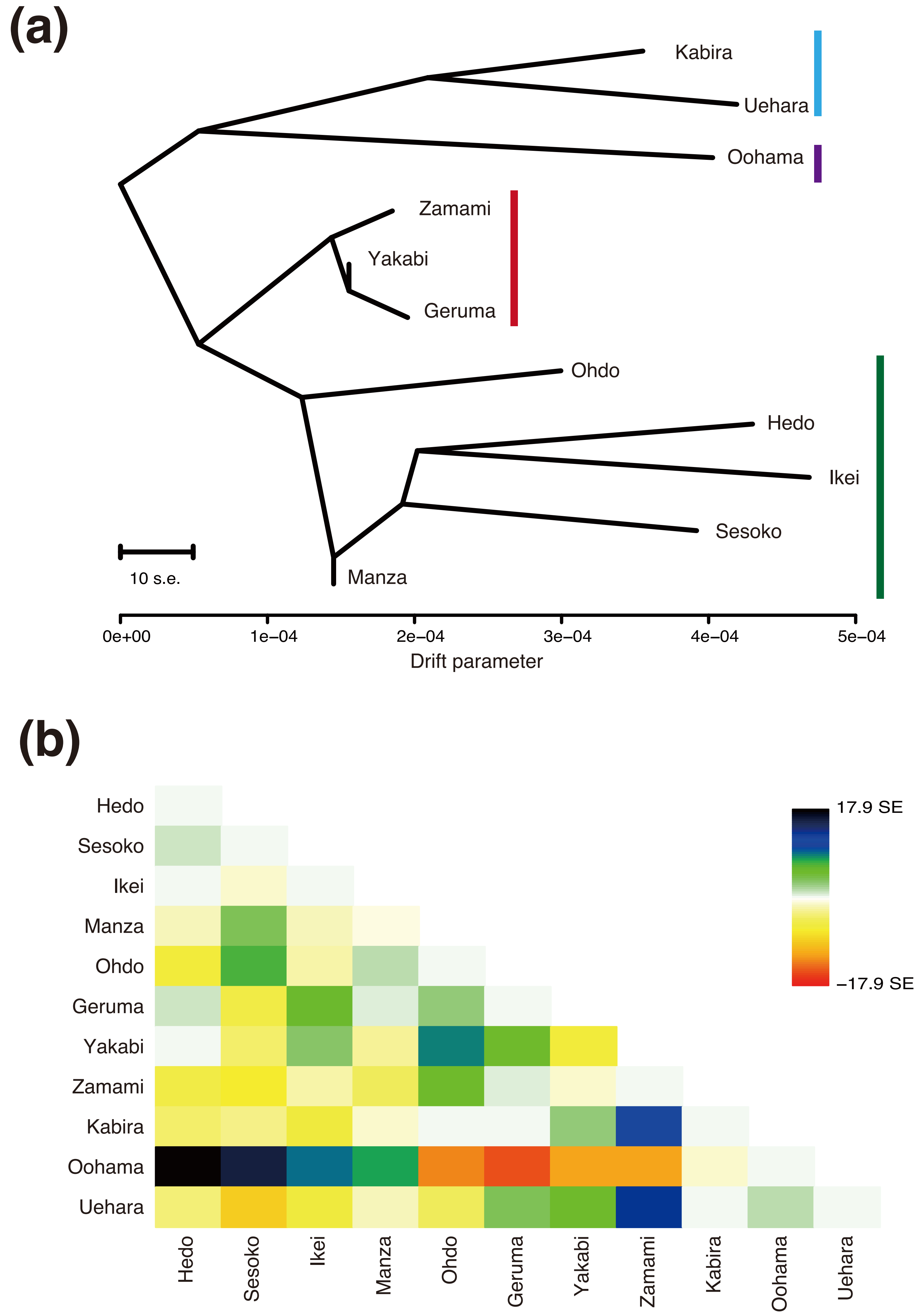

Supplementary Fig. 5

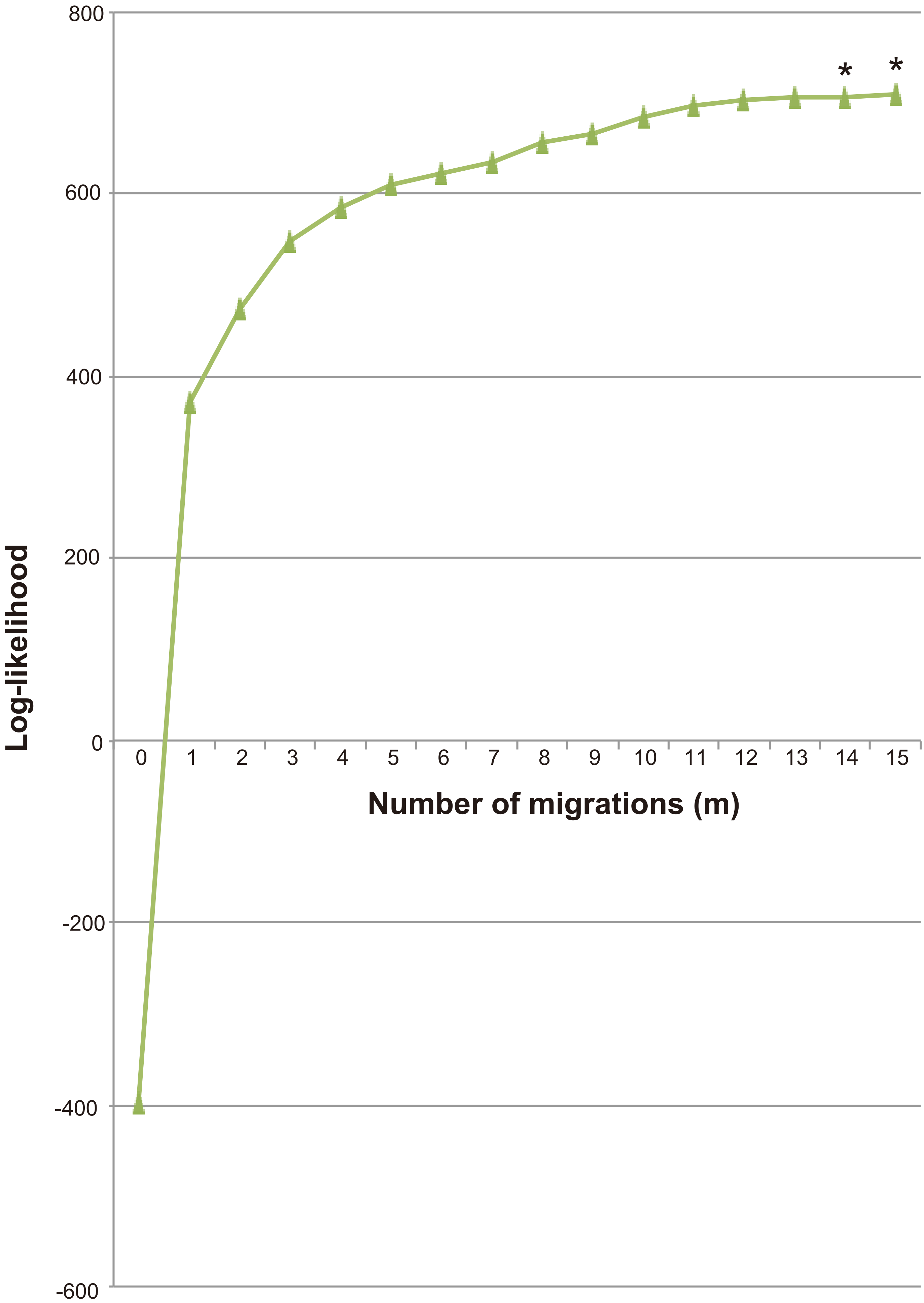

Supplementary Fig. 6

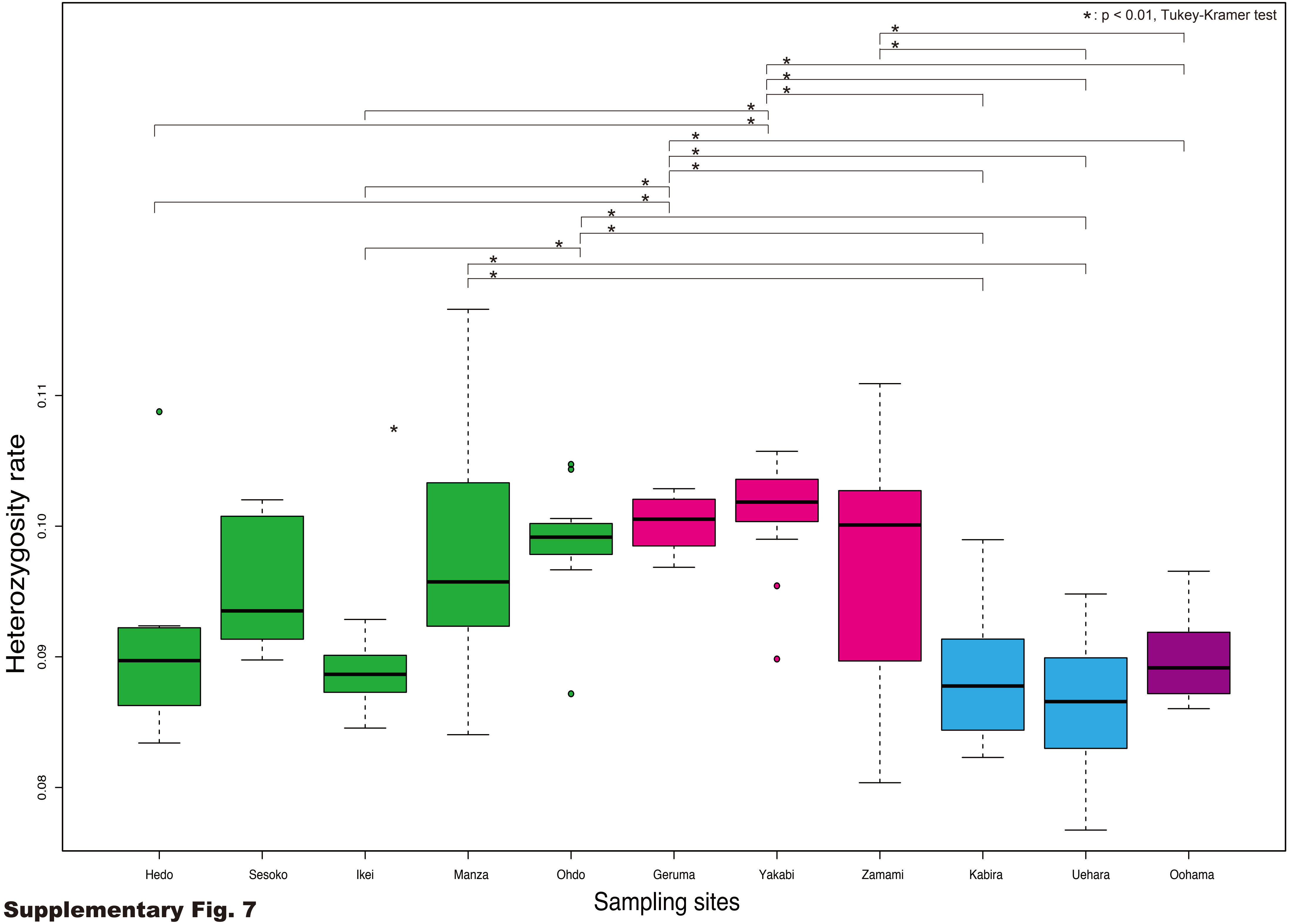

Supplementary Fig. 7

Supplementary Table 1. Summary of *Acropora digitifera* genome assembly version 1.1.

	Ver. 1 (Shinzato et al., 2011)*	Ver. 1.1 (This study)
Total assembly basepair	419 Mbp	447 Mbp
Number of Scaffolds	4,765	2,420
Maximum scaffold length (bp)	1,590,019	2,549,845
N50 length (bp)	191,489	483,559
Number of scaffolds longer than 1 Mbp	6	59
GC %	33.98	33.09
Gap %	12.96	15.24

* Shinzato et al. (2011). Using the *Acropora digitifera* genome to understand coral responses to environmental change. *Nature* 476, 320-323.

Supplementary Table 2. Mapping coverage of each individual.

Sample name	Coverage	SD
Hd1	6.3606	89.3299
Hd2	5.6974	28.8122
Hd3	4.4638	23.8887
Hd4	5.2785	31.211
Hd5	6.0081	22.733
Hd6	4.4947	34.272
Hd7	5.4441	33.7491
Hd8	6.8873	53.0772
Hd9	5.0283	31.8202
Ik1	5.9052	24.1078
Ik2	5.3453	20.4816
Ik3	4.9175	12.0784
Ik4	5.4435	14.9242
Ik5	5.0572	18.8035
Ik6	5.5356	21.8584
Ik7	4.4913	12.3866
Ik8	5.1384	22.0075
Ik9	4.6097	22.6955
Irm10	4.4736	26.3762
Irm17	4.4157	24.1778
Irm2	6.3191	39.5431
Irm21	6.2886	21.3364
Irm22	5.1876	22.0857
Irm23	4.7185	17.5663
Irm24	5.36	31.15
Irm25	5.305	23.9669
Irm26	5.8949	21.6899
Irm27	6.0486	24.6419
Irm3	5.685	44.4
Irm4	4.8595	28.4608
Irm5	4.823	33.0183
Irm7	4.898	35.4157
Irm9	5.3694	25.5019
IS1	5.378	34.6059
IS10	7.2266	29.677
IS2	5.4952	26.2356
IS3	4.8314	22.2159
IS4	5.5703	24.5537
IS5	5.7003	34.1984
IS6	5.0165	29.8529
IS7	6.205	35.6842
IS8	4.597	24.0946
IS9	5.7699	32.1219
Isy12	8.26	60.55
Isy15	6.36	48
Isy16	7	51.145
Isy17	6	40
Isy18	7.8	49.48
Isy21	4.554	14.94
Isy22	4.58	10.3
Isy23	5.38	17.6
Isy24	5.65	22.4

Isy25	4.2	16.12
Isy26	5.95	19.8
Isy27	4.66	16.5
Isy4	6.36	26.6
Isy7	7.9	40.61
Isy8	7.957	26.733
KrA1	7.7891	16.8909
KrA10	9.3605	25.7282
KrA11	6.9799	16.9296
KrA12	9.9389	28.7032
KrA13	9.3215	29.2653
KrA14	8.7348	25.1737
KrA15	7.4747	14.0994
KrA2	7.2758	15.5348
KrA3	8.7793	18.0328
KrA4	10.8014	42.956
KrA5	7.8821	27.2656
KrA6	10.3948	51.1178
KrA7	7.7349	21.0603
KrA8	7.0726	16.2489
KrA9	8.5935	24.9698
KrC1	8.9343	30.5972
KrC10	9.3455	55.0326
KrC11	11.8119	69.6765
KrC12	9.9486	41.8156
KrC13	11.9591	61.6358
KrC14	10.1248	58.5169
KrC15	9.8893	65.5252
KrC2	7.0252	33.8842
KrC3	9.2138	47.4095
KrC4	5.4491	11.8477
KrC5	8.8802	24.771
KrC6	8.0571	30.6705
KrC7	9.6861	38.1604
KrC8	11.4566	33.3997
KrC9	9.281	28.987
KrD1	3.765	32.162
KrD11	3.9	11.61
KrD13	4.22	35.716
KrD3	2.876	21.565
KrD8	2.9243	22.628
KrD9	3.176	25.642
KrE1	10.1789	35.8984
KrE10	5.0788	27.7967
KrE11	7.171	25.6827
KrE12	7.5259	25.8185
KrE13	5.9007	33.146
KrE14	5.7978	21.0957
KrE15	8.4677	19.5119
KrE2	10.6866	50.7823
KrE3	8.2071	27.619
KrE4	9.7364	36.2596
KrE5	9.1076	28.4391
KrE6	20.0578	103.0541
KrE7	3.6371	5.8318

KrE8	14.9095	79.6395
KrE9	4.9126	19.6334
mz1	10.6959	75.5944
mz10	5.4695	35.4351
mz11	5.0278	18.7131
mz12	5.9639	15.2008
mz2	9.8257	208.1963
mz3	6.4756	80.0516
mz4	10.691	66.9277
mz5	6.9743	39.5089
mz6	6.9991	44.3849
mz7	7.4523	32.4522
mz8	5.6297	30.8291
mz9	6.1119	28.5675
mzC1	8.35	29.78
mzC2	7.46	30.19
MzC3	4.44	22.18
MzC4	6.458	25.5
od1	10.6988	41.6719
od10	9.49	38.42
od11	9.37	30.46
od12	9.63	34.23
od13	9.42	39.9
od14	8.74	48.85
od15	8.76	27.57
od18	8.86	35.52
od19	9.04	32.02
od2	4.5628	46.7632
od22	8.75	29.05
od23	9.06	35.45
od24	8.28	43.34
od25	9.15	39.02
od26	8.95	41.78
od27	8.48	29.29
od3	11.1614	43.0636
od4	5.5975	47.0097
od5	6.2188	55.1213
od6	5.6517	37.8428
od7	9.3188	86.4964
od8	5.5412	96.2062
od9	4.8686	86.4572
ss1	9.8407	51.2597
ss2	10.3453	63.9256
ss3	7.2498	45.1932
ss4	6.4991	63.7103
ss5	5.9955	36.0256
ss6	6.4787	40.45
ss7	6.4503	25.1972
ss8	5.9552	42.6045

Abbreviations: Hd: Hedo, Ik: Ikei, Irm: Uehara, IS: Oohama, Isy: Kabira, KrA: Geruma, KrC: Yakabi, KrD: Aka, KrE: Zamami, mz: Manza, od: Ohdo, ss: Sesoko.

Supplementary Table 3. Three-population test for *A. digitifera* of Okinawa

Target	Source1	Source2	f3	std.err	Z score
Yakabi	Ohdo	Geruma	−8.54E−05	8.59E−06	−9.94898
Yakabi	Kabira	Geruma	−6.97E−05	9.10E−06	−7.66525
Yakabi	Oohama	Geruma	−7.31E−05	9.70E−06	−7.53858
Manza	Ohdo	Oohama	−9.16E−05	1.25E−05	−7.3152
Yakabi	Uehara	Geruma	−6.23E−05	9.41E−06	−6.62242
Yakabi	Zamami	Geruma	−4.80E−05	8.34E−06	−5.74926
Yakabi	Geruma	Sesoko	−6.23E−05	1.11E−05	−5.62397
Yakabi	Hedo	Geruma	−5.11E−05	1.03E−05	−4.95832
Yakabi	Geruma	Manza	−4.12E−05	9.63E−06	−4.27461
Yakabi	Ikei	Geruma	−4.38E−05	1.03E−05	−4.25703
Yakabi	Zamami	Ikei	−4.03E−05	1.02E−05	−3.9697
Manza	Uehara	Sesoko	−5.56E−05	1.44E−05	−3.85651
Yakabi	Zamami	Hedo	−3.95E−05	1.06E−05	−3.71388
Yakabi	Zamami	Ohdo	−3.11E−05	8.65E−06	−3.59165
Manza	Geruma	Sesoko	−4.92E−05	1.39E−05	−3.54186
Manza	Ohdo	Hedo	−3.38E−05	1.22E−05	−2.76861
Manza	Zamami	Sesoko	−3.15E−05	1.35E−05	−2.34103
Yakabi	Zamami	Manza	−2.18E−05	9.48E−06	−2.29607
Yakabi	Zamami	Sesoko	−2.52E−05	1.12E−05	−2.24909
Manza	Kabira	Sesoko	−2.93E−05	1.39E−05	−2.10726
Manza	Yakabi	Sesoko	−2.81E−05	1.34E−05	−2.08992
Manza	Oohama	Geruma	−2.58E−05	1.27E−05	−2.04074
Zamami	Kabira	Geruma	−1.84E−05	9.16E−06	−2.01258
Yakabi	Ohdo	Uehara	−1.91E−05	9.52E−06	−2.00447

p-value < 0.05 (Z score < -1.96) are shown

Supplementary Table 4. Migration weight and p-values inferred by *TreeMix* with 13 migration events allowed.

		Weight of the edge	p-values
Source	Sink		
Sesoko	Ohdo	0.382422	<2.22507e-308
Oohama	Hedo Ikei Manza Sesoko	0.50146	<2.22507e-308
Ohdo	Manza	0.126096	7.40816e-05
Ohdo	Yakabi	0.0749277	0.00814736
Ikei	Geruma Yakabi	0.202702	1.48395e-07

Bootstrap support more than 50% (see Supplementary Table 5) are shown.

Supplementary Table 5. Bootstrap percentages (100 replicates) for each migration edges (m= 3 to 14).

Number of migration events allowed in *TreeMix*		3	4	5	6	7	8	9	10	11	12	13	14
Source	Sink												
Seso	Ohdo	86	81	86	90	85	91	88	88	84	89	81	84
Ooha	Hedo<br>Ikei<br>Manz<br>Seso	99	97	98	92	94	85	90	87	83	78	79	76
Ohdo	Manz	32	60	67	73	63	76	67	71	69	73	68	76
Ohdo	Yaka	-	-	-	-	28	41	61	63	77	85	86	86
Ikei	Geru<br>Yaka	43	51	62	55	69	64	66	61	60	60	64	56
Ohdo	Yone	-	-	24	29	45	38	38	45	41	37	36	46
Seso	Manz	-	-	-	-	31	30	45	39	39	49	46	52
Hedo<br>Ikei<br>Manz<br>Seso	Ikei	-	-	-	22	28	28	32	32	34	29	29	33
Yone	Ohdo	-	-	-	-	-	25	30	34	29	34	31	26
Ooha<br>Ueha<br>Yone	Yaka	-	-	-	-	-	-	-	-	21	-	23	18
Seso	Yone	-	-	-	-	-	-	-	-	-	30	41	48
Ooha	Hedo	-	-	-	-	-	-	27	-	-	-	26	23
Yone	Manza	-	-	-	-	-	-	-	-	26	-	29	-
Ueha	Manz	-	-	-	-	-	-	-	-	-	22	29	47
Yone	Zama	-	-	-	-	-	-	-	-	-	-	30	32
Ueha	Ooha	-	-	-	-	-	-	-	-	-	-	26	20

"-" represents bootstrap support is lower than 20 or no migration edge detected.
